# Temporary Pacing with Active-Fixation Leads: Clinical and Economic Impact Versus Conventional Temporary Transvenous Pacing

**DOI:** 10.3390/jcm15010125

**Published:** 2025-12-24

**Authors:** Raimundo Vicente-Miralles, Nuria Rivas-Gándara, José Luis Antón Pascual, Raquel Adeliño, Raquel Ajo Ferrer, José María Núñez Martínez, Manuel Solera Suárez, Alicia Ibáñez Criado, Amaya García-Fernández, Juan Miguel Ruiz Nodar, Vicente Bertomeu-Gonzalez, Juan Gabriel Martínez Martínez

**Affiliations:** 1 Intensive Care Unit, Dr. Balmis University General Hospital, 03010 Alicante, Spain; vicente_rai@gva.es (R.V.-M.); anton_jospas@gva.es (J.L.A.P.); 2 ISABIAL—Instituto de Investigación Sanitaria y Biomédica de Alicante, 03010 Alicante, Spain; ajo_raq@isabial.es (R.A.F.); aliciaicriado@gmail.com (A.I.C.); ama_garcia@hotmail.com (A.G.-F.); ruiz_jmi@gva.es (J.M.R.N.); martinez.juangabriel@gmail.com (J.G.M.M.); 3 Cardiology Department, Vall d’Hebron University Hospital, 08035 Barcelona, Spain; nuria.rivas@vallhebron.cat (N.R.-G.); adelino.raquel@gmail.com (R.A.); 4 Ciber Cardiovascular, 28029 Madrid, Spain; 5 Cardiology Department, Dr. Balmis University General Hospital, 03010 Alicante, Spain; 6 Intensive Care Unit, Vinalopó University Hospital Elche, 03293 Elche, Spain; josemarianuma@gmail.com; 7 Intensive Care Unit, Francesc de Borja Hospital, 46702 Gandía, Spain; manuuci@gmail.com; 8 Department of Clinical Medicine, Miguel Hernandez University, 03202 Alicante, Spain; 9 Cardiology Department, Hospital Clinica Benidorm, 03501 Benidorm, Spain

**Keywords:** temporary pacemaker, temporary–permanent pacemaker, temporary pacemaker with active-fixation leads, semipermanent pacemaker, external permanent pacemaker

## Abstract

**Background/Objectives**: The use of temporary pacemakers is increasing every year. Conventional temporary pacemakers are connected to the myocardium via a passive-fixation lead (temporary pacing with passive fixation leads; TPPF), which compromises their effectiveness and safety. Off-label active-fixation systems (temporary pacing with active fixation leads; TPAF) are a safer alternative. The main objective of this study was to assess the clinical and economic impact of TPAF versus TPPF. **Methods**: We conducted a literature search based on the clinical outcomes of both pacing techniques. We then carried out a descriptive comparative analysis and extrapolated the results to the Spanish, European, and global populations. **Results**: Of the 1015 articles located, the analysis included five articles from ECTSF and eight from ECTEFA, prospective and focused on the recording of complications. It is estimated that the implementation of ECTEFA as the first option for ECT would lead to a 94.7% reduction in complications. In economic terms, it would mean a 55.72% reduction in the cost of the procedure. **Conclusions**: TPAF leads to considerable clinical improvement compared with TPPF. Furthermore, while the price of TPAF doubles the procedural cost, the reduced cost of hospital stays and treating complications means the active-fixation systems could substantially reduce the overall cost of temporary pacing for healthcare systems.

## 1. Introduction

Heart rhythm disturbances are a common reason for consultation and hospital admission and can be life-threatening. Some people with bradycardia may require temporary pacemaker implantation to ensure a stable rhythm. In temporary pacing, a lead is threaded through a vein to the ventricle to detect the patient’s heart rhythm and, if needed, stimulate myocardial contraction at a determined frequency. This is a common emergency procedure in many critical care units [1].

The purpose of temporary pacing is to manage symptomatic acute bradycardia, normally caused by degenerative disease of the cardiac conduction system, overdose of negative chronotropic drugs [2], acute myocardial infarction [3], conduction abnormalities in people with infective endocarditis [4], or electrolyte disorders [5]. Temporary pacing is also commonly used in the perioperative management of people undergoing cardiac surgery, especially valve replacement [6]. New techniques implemented in the field of interventional cardiology require pre- and post-procedural temporary cardiac pacing. These include transcatheter aortic valve implantation (TAVI) and alcohol septal ablation [7,8].

Currently, the only certified system for transvenous temporary pacing uses bipolar passive-fixation leads (with no mechanism for active fixation to the myocardium) and a reusable external pulse generator weighing around 800 g. Patients with this type of temporary pacemaker must remain on bed rest with continuous electrocardiographic monitoring. Despite these precautions, complication rates can exceed 35% [9]. The most common complications are associated with the procedure and include lead displacement resulting in loss of capture and need for repositioning, cardiorespiratory arrest, and heart perforation, sometimes requiring emergency surgery [10,11].

Proposed alternatives for reducing the complications and increasing the effectiveness of temporary pacing include the use of bipolar active-fixation leads with a reusable permanent pulse generator [12]. The latest European Society of Cardiology clinical practice guidelines on cardiac pacing recommend these active-fixation systems for long-term temporary transvenous pacing (class of recommendation IIa, level of evidence C) [13].

This study aimed to evaluate the clinical and economic impact of temporary pacing with active-fixation leads (TPAF) versus temporary pacing with passive-fixation leads (TPPF), to generate evidence on the use of safer and more effective devices for expanding clinical indications.

## 2. Materials and Methods

Objectives. The main objective of the study was to evaluate the safety and effectiveness of off-label active-fixation systems with a reusable permanent pulse generator (TPAF) versus certified conventional transvenous temporary pacemakers with bipolar passive-fixation leads (TPPF). Our secondary objective was to perform an economic analysis of TPAF versus TPPF systems.

Study design. We designed a descriptive study to evaluate reported complication rates of the two strategies. With the results, we estimated the difference in complications and costs between the two strategies in the Spanish, European, and global context.

To this end, we conducted a bibliographic search in PubMed, Google Scholar, and Cochrane Library for publications related to TPPF and TPAF, using the key words “Temporary Transvenous Cardiac Pacing”, “Cardiac Pacing”, “Temporary Cardiac Stimulation”, “Temporary Pacemaker”, “External Pacemaker”, “Temporary Permanent Pacemaker”, “External Permanent Pacemaker”, “Active-Fixation Temporary Pacemaker”, “Semipermanent Pacemaker”, and “Prolonged Temporary Transvenous Pacemaker”. We reviewed full-text articles published in any language from 1994 (last 30 years) that described complications of TPPF or TPAF in people aged 18 years or older. First, we screened the abstracts and eliminated studies whose objective was unrelated to our primary objective. Next, we reviewed the methods of the remaining studies and discarded studies with a retrospective design. This strict exclusion criteria was necessary because retrospective analyses often rely on clinical records where underreporting of non-major complications (e.g., transient lead displacement or bradycardia) is common. By exclusively focusing on prospective studies designed to record complications, we ensured the complication rates used in our model were based on the most accurate and robust clinical data, despite the resulting low number of included articles (five TPPF and eight TPAF).

For our safety objective, we collected the absolute number and rate of the most frequent temporary pacemaker-related complications: lead displacement, heart perforation/rupture, cardiac tamponade, pericardial effusion, death, bradycardia, ventricular arrhythmias, infection, and non-cardiac hemorrhage. For our effectiveness objective, we collected the number of pacemaker failures. In all cases, we collected the duration of temporary pacing.

For the economic analysis of TPAF versus TPPF, we collected costs associated with the procedures, hospital stays, the medical devices, and treating complications.

We then extrapolated our results to the European Union population and the global population. The global population included the 100 countries with the best healthcare systems according to the Healthcare Access and Quality Index (HAQ Index) [14], because we only wanted to include countries where temporary pacing is a common procedure. We extracted population data for each country from the public EUROSTAT website [15].

We analyzed the data using the spreadsheet software Excel (Microsoft Corp., Redmond, WA, USA) then generated tables of the results.

Calculation of economic costs of temporary pacing. Our cost calculation was divided into three general concepts: (1) cost of the temporary pacemaker implantation procedure, including personnel and medical devices; (2) cost of hospital stay; and (3) cost of treating temporary pacemaker-associated complications. We based our estimates on costs published by the Spanish Health System [16].

Both procedures have similar personnel costs. The cost of medical devices included single-use consumables but not reusable generators, which are used in multiple patients and thus have a negligible per-patient cost. To calculate the cost of hospital stays, we used an average temporary pacing duration of seven days, based on publications about active-fixation systems in the general population [12,17]. Studies of TPAF do not limit the duration of therapy due to lack of safety or effectiveness: the device remains in place for the time needed to make decisions. People with TPPF have to stay in the critical care unit throughout therapy, whereas we counted only one day in the critical care unit for people with TPAF, followed by six days in a ward with telemetry.

## 3. Results

### 3.1. Results of the Bibliographic Search

**Figure 1:**
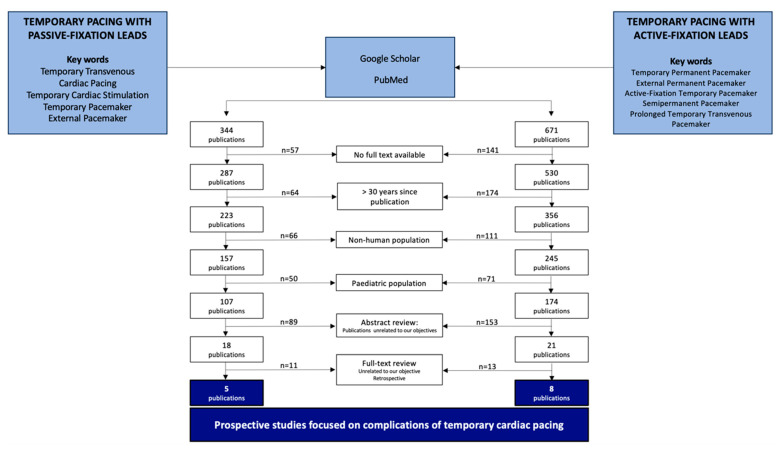
Flowchart illustrating the bibliographic search and selection of articles.

The bibliographic search returned 344 unique studies on TPPF and 671 unique studies on TPAF. We identified no systematic reviews or meta-analyses related to our study objective. After applying the relevant filters (full text available, publication from 1994, patients aged 18 years and older), we had 107 TPPF articles and 174 TPAF articles. During the abstract review, we eliminated single case reports and articles whose main or secondary objective was unrelated to the safety and effectiveness of temporary pacing, leaving 18 TPPF publications and 21 TPAF publications. We then reviewed the methods and eliminated retrospective studies, as these may have incomplete data (not all complications recorded in clinical records). The final analysis included five TPPF articles and eight TPAF articles. Twelve publications described prospective case series, and one described a prospective, comparative, non-randomized study evaluating clinical differences between the strategies [11] (Figure 1).

### 3.2. Epidemiology of Temporary Pacing

#### 3.2.1. Population

According to data obtained from the public website EUROSTAT [15], Europe Union has a population of 449 million inhabitants. The 100 countries with the best healthcare systems according to the HAQ Index have a combined population of 3.736 billion people.

#### 3.2.2. Number of Temporary Pacemaker Implantations

People who have temporary pacemaker implantations can be divided into two large groups based on the indication for therapy. The first group covers all temporary pacing indications except TAVI procedures. The incidence of temporary pacemaker implantations for these indications is 11.32 per 100,000 inhabitant-years [18]. The second group includes people undergoing TAVI. The incidence of TAVI per 100,000 inhabitant-years varies considerably between countries depending on the rate of adoption of the procedure, from 14.07 in low rate countries as Spain [8] to 22.23 in the USA [19] or 23.36 in Germany [7]. For this study, we calculated the mean rate across countries, which was 17.71 implants per 100,000 inhabitant-years.

**Figure 2:**
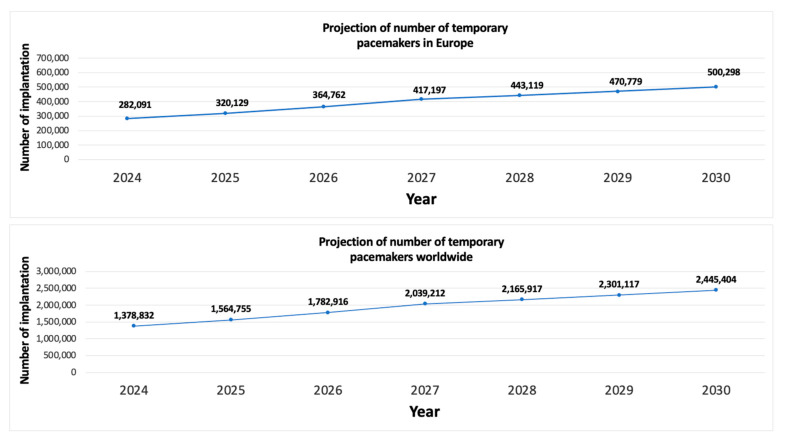
Predicted number of temporary pacemaker implants.

In 2024, an estimated 165,771 implants will have been performed in Europe and 1,378,832 worldwide. Using these data, we applied annual growth rates to estimate the number of procedures up to 2030, according to the different indications for temporary pacing. In the first group (all indications except TAVI), the expected annual growth rate is 3.12%, associated with population aging and the likely increase in cardiovascular diseases [20]. The expected growth rate in the second group is 7.2%, given the growing preference for TAVI over open valve surgery [21]. With these predictions, we estimated the number of temporary pacemaker implants in 2030 would be 500,000 in Europe and 2.4 M worldwide (Figure 2).

### 3.3. Clinical Impact of the Different Temporary Pacing Systems

Studies evaluating TPPF have reported a higher rate of complications despite a shorter duration of therapy (0.35 complications/procedure over 3.7 days) compared with TPAF (0.03 complications/procedure over 11.8 days) (Table 1). Based on these rates, we estimated the number of temporary pacemaker-associated complications in 2024 would be 93,375 in Europe and 776,288 globally if all temporary pacing procedures used passive-fixation systems, versus 4946 in Europe and 40,840 globally if all temporary pacing procedures used active-fixation systems (Table 2).

Using our estimates of the annual number of implantations per population and the differing rates of complications between the two procedures, we estimated that TPAF versus TPPF would lead to 88,465 fewer complications in Europe, and 716,352 fewer complications worldwide (94.7% reduction) (Table 3).

### 3.4. Economic Impact of the Different Cardiac Pacing Systems

#### Cost of Temporary Cardiac Pacing

The cost of temporary cardiac pacing depends on the type of material used, the duration of therapy, the unit where the patient receives care during their hospital stay, and the cost of treating complications related to the procedure. We based our estimates on costs published by the Spanish Health System [16].

The staff cost assigned to a temporary pacemaker implantation is EUR 539.03. The material used for TPPF has an estimated cost per procedure of EUR 229.83, which includes EUR 205.00 for the bipolar passive-fixation lead (Ref 007406P, Bard Medical, 1 Becton Drive Franklin Lakes, 07417-1880, New Jersey, NJ, USA) and EUR 24.83 for the 7.5-FR percutaneous introducer sheath (Ref SI-09880-LF, Arrow Medical Limited, Hatton Gardens, Industrial Estate, Kington Herefordshire HR5 3RB, Kington, UK).

The material used in TPAF amounts to EUR 926.90: EUR 383.90 for a bipolar active-fixation lead (SOLIA S60, Ref 466706, BIOTRONIK SE & Co. KG, Woermannkehre 1, 12359 Berlin, Germany); EUR 43.00 for a 7.5-FR peelable percutaneous introducer sheath (Ref 405153, Abbott Cardiovascular, 5050 Nathan Lane North, Plymouth, MN 55442, USA), and EUR 500 for a dedicated fastening system for active-fixation temporary pacemakers (e.g., KronoSafe^®^ System, ICU Medical Technologies S.L. Santa Anna 21th, 03204, Elche, Spain), included here as an example of an external fixation solution. We excluded the cost of the generator from our calculations as it used in multiple patients over several years, and its in-hospital cleaning and sterilization do not represent a significant additional expense.

Depending on the temporary pacing system used, patients receive therapy in different units of the hospital, which also affects the cost of care. Those who receive TPPF require continuous monitoring due to the risk of lead displacement, often staying in a Critical Care Unit (ICU) or transferred to a Medical Intermediate Care Unit once stable. Those who receive TPAF can generally stay in a cardiology ward with telemetry monitoring. Based on an average duration of seven days of temporary pacing, the cost associated with TPPF is calculated as one day in critical care (EUR 1365.29) and six days in an Intermediate Care Unit (EUR 920 per day), totaling EUR 6885.29 per patient. This is compared with EUR 3453.47 per patient among those who receive TPAF (one day in critical care and six days in a ward).

We used the cost of treating temporary pacemaker-related complications, together with their incidence rates, to estimate the average additional cost per procedure. The estimated mean cost of treating TPPF-associated complications was EUR 3827.26 per procedure, compared with EUR 191.42 per procedure for treating TPAF-associated complications (Table 4). The estimated total per-procedure cost of temporary pacing—including medical devices, personnel, hospital stay and treating complications—was EUR 115442.18 for TPPF and EUR 5110.82 for TPAF (Table 5). Our economic analysis suggests that using TPAF instead of TPPF reduces the overall cost of temporary pacing by 55.72%, which translates to an annual saving of EUR 1.076 billion in Europe and EUR 8.948 billion worldwide (Table 6).

## 4. Discussion

Numerous publications have shown that temporary pacing systems with active-fixation leads are sufficiently safe and effective for use outside the critical care unit [12,17,23], and favorable results have even been reported outside the hospital setting [30]. The average duration of cardiac pacing varies considerably between studies on TPPF (3.7 days) and TPAF (11.8 days), mainly because of differences in the indication for pacing. Studies of TPAF have included people who needed a longer duration of pacing, for reasons such as permanent pacemaker-associated infection. In contrast, because of the high frequency of associated complications [10,11], and the need for patients to stay in the critical care unit, clinicians are more likely to remove TPPF systems early, often too early.

**Figure 3:**
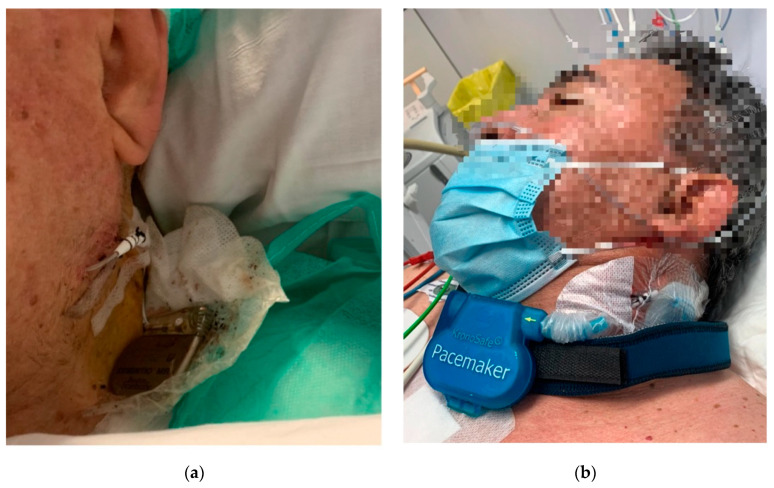
(**a**) Temporary-permanent pacemaker. Assessment at 24 h post-implant. (**b**) Example of a dedicated external fastening system for active-fixation temporary pacemakers.

One of the limitations of TPAF is the lack of certified devices specifically designed for this purpose. The off-label use of permanent pacemakers externalized for temporary cardiac pacing has improved clinical results and recently the European Society of Cardiology recommends these systems in their last cardiac stimulation guidelines [13]. However, there are still risks for the patient, such as intravascular infection or accidental detachment of the generator (Figure 3a). Solutions such as specialized external fastening systems could address the risk of accidental detachment associated with externalized permanent pacemakers (Figure 3b) [12].

Because TPAF systems are safe and effective, the duration of temporary pacing can be extended to better suit the needs of the patient and the healthcare system. Kordoni and colleagues reported optimal functional outcomes in a patient with autonomic dysfunction secondary to Miller-Fisher syndrome who was treated with TPAF for five weeks [31]. With TPAF systems, clinicians have more time for clinical decision-making and can avoid unnecessary permanent pacemaker implantations. Henry D and colleagues recognize that external pressure on clinicians to reduce the length of hospital stays and mobilize patients early after TAVI may lead to unnecessary indications for permanent pacemaker implantation [32]. Reported rates of atrioventricular conduction recovery after TAVI are as high as 50%. Including active-fixation systems to assist the TAVI procedure and maintain the temporary pacemaker during the patient’s hospital stay (median of seven days) [33] could optimize decision-making.

As with any external device, there is a risk of accidental pulling and detachment of the system, which would put the patient’s life at risk in this case. External fastening solutions could help standardize the use of this material, potentially increasing treatment safety [12].

Regarding the economic impact of TPAF, we estimated considerable savings for healthcare systems owing to the lower cost of hospital stays in the cardiology ward versus the critical care unit (Table 6). In addition, providing temporary pacing in a ward reduces the caseload of high-complexity units. Lastly, because patients on TPAF systems can receive temporary pacing for longer, clinicians can optimize indications for permanent pacemaker implantation, possibly reducing the number of unnecessary procedures. The cost of the implantation, follow-up, and management of complications over the first year can be up to EUR 7000, then EUR 500 per year for the remainder of treatment [16,34].

The costs associated with temporary pacing vary across countries and healthcare systems. Nonetheless, we estimated that the use of active-fixation systems could reduce the overall cost of temporary pacing by 55%, mainly owing to the reduced cost of hospital stays and treatment of complications.

The excellent clinical results published to date with active fixation leads and their expected associated economic benefits were not reflected in real practice with The Tempo Lead (Merit Medical Systems Inc., 1600 Wert Merit Parkway, South Jordan 84095, UT, USA) [35]. The lack of clinician adoption led to the discontinuation of the distally stabilized lead. Not only all of these aspects represent a comparative benefit of TPAF but also, the possibility of performing temporary cardiac stimulation at home when used with a secure external fixation system. Out-of-hospital temporary cardiac stimulation would allow for an increased duration of patient’s heart rhythm evaluation and to more accurately determine the need for a permanent pacemaker.

Our study has some limitations, such as its descriptive design, the substantial variability between the reported clinical results of TPPF (possibly depending on each center’s experience with this treatment), the scarcity of prospective studies comparing the two systems (TPPF vs. TPAF), the age of the studies (most from the 2000s), and possible nonpublication of studies with unfavorable results (publication bias). A significant limitation of our economic analysis is the extrapolation of costs. The cost figures for personnel, hospital stays, and complication treatments were sourced exclusively from the Spanish Health System. While this allowed for a direct comparative assessment, we acknowledge that healthcare costs and resource allocation vary considerably across European and global populations. Therefore, the financial savings estimated in Table 5 and Table 6 should be interpreted as theoretical projections and indicators of magnitude, rather than precise costs applicable to all healthcare environments globally.

The duration of temporary cardiac pacing between the TPPF and TPAF groups also differed significantly. This may be primarily due to the difference in the indication for pacing and the need to make hasty decisions in the TPPF group due to its lack of safety. We do not consider that this difference does not allow us to compare both procedures, and furthermore, despite the longer times in the TPAF group, the safety and effectiveness results are better.

## 5. Conclusions

Temporary cardiac pacing is an increasingly common procedure, especially in relation to new interventional cardiology techniques. The use of active-fixation systems for temporary cardiac pacing increases the safety and effectiveness of the procedure. The direct and indirect cost of temporary cardiac pacing is high but could be reduced through active-fixation systems.

## 6. Patents

The KronoSafe System^®^ in patented under no. ES 2 804 080 A1; ICU Medical Technologies S.L., 3 February 2021.

## Figures and Tables

**Table 1:** Publications included number of patients, duration of therapy, and number and rate of complications.

Temporary Pacing with Passive-Fixation Leads				
Study	No. of Patients	Mean Duration of Pacing	No. of Complications	Rate of Complications
Betts et al. 2003 [10]	144	2 days	81	0.56
Braun et al. 2006 [11]	26	7.7 days	24	0.96
Mortera et al. 2013 [22]	266	Not reported	45	0.16
Murphy et al. 1996 [23]	194	Not reported	77	0.39
Ferguson et al. 1997 [24]	40	1.5 days	8	0.20
Total	670	3.7 days	235	0.35
Temporary Pacing with Active-Fixation Leads				
Study	No. of Patients	Mean Duration of Pacing	Number of Complications	Rate of Complications
Braun et al. 2006 [11]	23	8.2 days	1	0.04
Vicente-Miralles et al. 2022 [12]	20	7.6 days	0	0
Kawata et al. 2013 [25]	23	19.4 days	1	0.04
Lepillier et al. 2012 [26]	8	8 days	0	0
Chihrin et al. 2006 [27]	20	2 days	1	0.05
Zei et al. 2006 [17]	62	7.5 days	0	0
Rastan et al. 2005 [28]	10	13.5 days	0	0
Lever et al. 2003 [29]	20	28 days	2	0.10
Total	186	11.8 days	5	0.03

**Table 2:** Estimated number of complications during 2024 for temporary pacing with passive-fixation leads (TPPF) and with active-fixation leads (TPAF).

	TPPF			TPAF		
	Rate	Europe	Global	Rate	Europe	Global
Lead detachment	0.21	34,749	288,653	0.01	1937	16,111
Heart perforation	0.03	4375	36,386	0.00	0	0
Death	0.07	12,272	102,073	0.00	0	0
Bradycardia	0.15	24,387	202,847	0.00	0	0
Ventricular arrhythmia	0.04	5830	48,496	0.00	0	0
Infection	0.04	6116	50,874	0.02	2973	24,729
Non-cardiac bleeding	0.03	5646	46,959	0.00	0	0
TOTAL	—	93,375	776,288	—	4946	40,840

**Table 3:** Reduction in complications associated with temporary pacing with active-fixation leads (TPAF) versus temporary pacing with passive-fixation leads (TPPF).

	No. of Complications		
	TPPF	TPAF	Δ Complications
Europe	93,375	4946	88,789
Worldwide	776,288	40,840	735,448
**Reduction in complications**		**94.7%**	

**Table 4:** Cost of complications associated with temporary pacing with passive-fixation leads (TPPF) and with active-fixation leads (TPAF).

		TPPF		TPAF	
Complication	Cost per Event (EUR)	Rate	Cost per Procedure (EUR)	Rate	Cost per Procedure (EUR)
Lead detachment	539.03	0.209	112.84	0.012	6.30
Heart perforation	31,947.73	0.026	843.07	0	—
Bradycardia	16,901.62	0.147	2486.49	0	—
Ventricular arrythmia	114.11	0.035	4.01	0	—
Infection	10,322.09	0.037	380.85	0.018	185.12
**TOTAL**	**—**	**—**	**3827.26**	**—**	**191.42**

**Table 5:** Cost of temporary pacing with passive-fixation leads (TPPF) and with active-fixation leads (TPAF).

	Cost (EUR)	
	TPPF	TPAF
**Personnel**	**539.03**	**539.03**
**Medical devices**	**229.83**	**926.90**
Pacing lead	205.00	383.90
Introducer sheath	24.83	43.00
Pacemaker fastening system	—	500
**Hospital stay**	**6885.29**	**3453.47**
Critical care	1365.29 (1 day)	1365.29 (1 day)
Intermediate care	5520.00 (6 days)	
Cardiology ward	—	1861.02 (6 days)
24-h telemetry	—	227.16 (6 days)
**Complications (mean cost)**	**3827.26**	**191.42**
**TOTAL**	**11542.18**	**5110.82**
**Cost reduction**	**55.72%**	

**Table 6:** Annual cost of temporary pacing procedures in Europe and worldwide; and saving in the three populations with use of temporary pacing with active fixation leads (TPAF) versus temporary pacing with passive-fixation leads (TPPF). Modeled savings based on Spanish standard costs.

		Europe	World
Number of procedures	—	165,771	1.278832
Cost of procedure (EUR)	TPPF	83.355584	743.231918
	TPAF	83.355584	743.231918
Cost of material (EUR)	TPPF	38.099167	316.897003
	TPAF	63.490323	528.092731
Cost of hospital stay (EUR)	TPPF	1141.610729	9494.220128
	TPAF	572.485449	4761.755625
Cost of treating complications (EUR)	TPPF	634.448709	5277.146718
	TPAF	31.732278	263.940041
**Total saving with TPAF (EUR)**		**1.076 billion**	**8.948 billion**

## Data Availability

The original contributions presented in this study are included in the article. Further inquiries can be directed to the corresponding author.

## Abbreviations

The following abbreviations are used in this manuscript:

HAQ Index	Healthcare Access and Quality Index
TAVI	Transcatheter aortic valve implantation
TPAF	Temporary pacing with active fixation leads
TPPF	Temporary pacing with passive fixation leads
